# Anaphylaxis to *Agaricus bisporus* ingestion

**DOI:** 10.31744/einstein_journal/2020RC5478

**Published:** 2020-11-18

**Authors:** Inês Machado Cunha, Maria Luís Marques, Carmo Abreu, Borja Bartolomé, Eva Gomes

**Affiliations:** 1 Centro Hospitalar Universitário do Porto Immunoallergology Department Porto Portugal Immunoallergology Department, Centro Hospitalar Universitário do Porto, Porto, Portugal.; 2 Centro Hospitalar de Trás-os-Montes e Alto Douro Immunoallergology Department Vila Real Portugal Immunoallergology Department, Centro Hospitalar de Trás-os-Montes e Alto Douro, Vila Real, Portugal.; 3 Roxall España Research and Development Department Bilbao Spain Research and Development Department, Roxall España, Bilbao, Spain.

**Keywords:** Anaphylaxis, Agaricales, Immunoblotting

## Abstract

A 33-year-old male with house dust mite allergic rhinitis and asthma reported an episode of facial and lip angioedema, dyspnea, cough and dysphagia at the age of 25, minutes after eating a mushroom ( *Agaricus bisporus* ) pizza. He denied any drug intake, hymenoptera stings or other possible triggers, and no identifiable cofactors were present. Since then he avoided all types of mushrooms, however an accidental contact occurred with mushroom sauce that resulted in angioedema of the lip within minutes. The allergy workup included measurements of total IgE and specific IgE to mushroom, and skin prick test to aeroallergens sources, possible food allergen sources and mushroom extract, a prick to prick test with raw and cooked *A. bisporus* , in addition to a SDS-PAGE and immunoblotting assay. The study revealed a specific IgE to mushroom of 0.76kUA/L positive skin prick test to mushroom extract, and prick to prick test positive to white and brown *A. bisporus* (raw and cooked). The immunoblotting identified two IgE binding proteins with 10kDa and 27kDa. We report a case of *A. bisporus* anaphylaxis probably due to primary mushroom sensitization. We detected two IgE-reactive proteins with 10kDa and 27kDa as possible culprit allergens.

## INTRODUCTION

Anaphylaxis is a serious allergic reaction with a rapid onset and potentially fatal outcome.^(^^1^^)^

In adults the main anaphylaxis triggers are food, insect stings and drugs.^(^^2^^)^

Concerning food allergens, the main identified triggers are peanut, fish, shellfish, tree nut and fresh fruits, especially in pollen allergic patients; however we must be aware that the implicated allergens can change with different eating patterns.^(^^3^^,^^4^^)^ In a suspected food allergy, a clinical history with a food diary is extremely important to identify unusual food allergens.

Species in the Fungi kingdom can cause different types of allergic symptoms. Allergens from mold spores are mainly associated with airborne respiratory allergies. Mushroom species can be implicated in contact dermatitis and also cause digestive symptoms, accounting for 1% of mushroom allergy.^(^^5^^,^^6^^)^

*Agaricus bisporus* is the largest cultivated mushroom, accounting for 38% of the world production.^(^^7^^,^^8^^)^ Only few cases of allergy due to ingestion of *A. bisporus* have been described. The majority of cases reporting mushroom allergy due to ingestion refer to the species *Boletus edulis* , *Boletus badius* , *Lentinus edulus and Tricholoma matsutake.* ^(^^9^^,^^10^^)^

We report a case of anaphylaxis to *A. bisporus* in a patient without any other sensitization to molds, in which ingestion appears to be the primary route of sensitization.

## CASE REPORT

A 33-year-old man with allergic rhinitis and asthma under treatment with inhaled and nasal corticosteroids, and with a known allergy to *Dermatophagoides pteronyssinus* and *Dermatophagoides farinae* .

He experienced an anaphylactic reaction at the age of 25 years, with facial and lip angioedema, dyspnea, cough and dysphagia minutes after ingestion of a mushroom pizza. No other suspected triggers were present, such as drug intake or stings. He also denied physical exercise or alcohol intake in the hours before the episode. He stopped eating mushrooms and any food containing mushrooms.

The patient reported two posterior episodes of lip angioedema, without any other symptoms after accidental contact with mushroom sauce.

Measurements of serum total IgE (ImmunoCAP™, Phadia, ThermoFisher Scientific, Uppsala, Sweden) and specific IgE (ImmunoCAP™, Phadia, ThermoFisher Scientific, Uppsala, Sweden) to airborne allergenic sources ( *D. pteronyssinus* , *D. farina* e, *Lepidoglyphus destructor* , cat and dog dander) molds ( *Aspergillus fumigatus and Alternaria alternata* ) and mushroom were carried out.

We also performed skin prick tests with commercial extracts (LETI Laboratories, Madrid, Spain) of *D. pteronyssinus* (100HEP/mL); *D. farinae* (100HEP/mL); *L. destructor* (10HEP/mL); pollens from *Olea europaea* (30HEP/mL), *Corylus avellana* (30HEP/mL), and *Platanus occidentalis* (30HEP/mL); cat and dog dander (30HEP/mL); *A. fumigatus* (150ug protein/mL); *A. alternata* (30HEP/mL); *Cladosporium herbarum* (150ug protein/mL); *Candida albicans* (125ug protein/mL); pollen from grass mixture (30HEP/mL); cow's milk (1,600ug protein/mL), egg (1,400ug protein/mL); wheat flour (1,900ug protein/mL); tomato (10HEP/mL) and mushroom (10HEP/mL). Histamine was used as positive control (10mg/mL).

Besides skin testing with commercially available extracts, a prick to prick test with raw and cooked *A. bisporus* was also carried out.

A sodium dodecyl sulfate-polyacrylamide gel electrophoresis (SDS-PAGE) immunoblotting assay was performed to assess the molecular mass of the IgE-binding proteins from *A. bisporus* .

## RESULTS

Total IgE was 240kU/L and specific IgE was positive to mushroom (0.76kU_A_/L), *D. peteronyssinus* (30.90kU_A_/L) and *L. destructor* (2.33kU_A_/L).

Skin prick tests were positive to extracts from mushroom (7mm), *D. pteronyssinus* (10mm), *D. farinae* (8mm), *L. destructor* (7mm).

**Figure 1:**
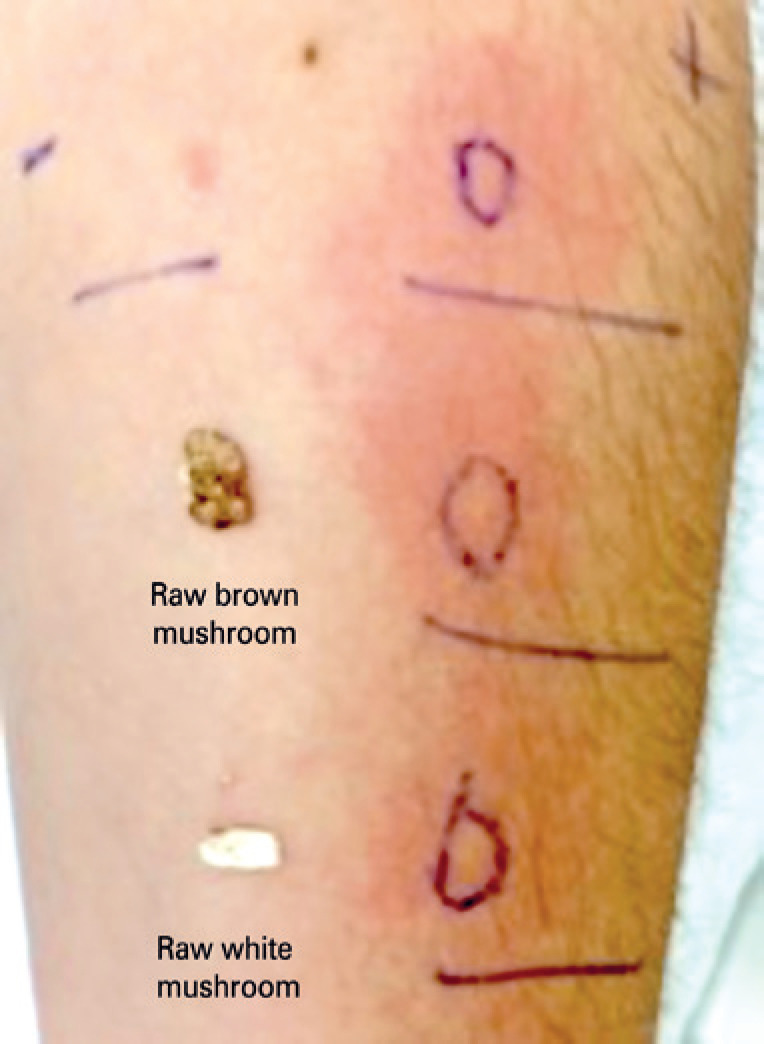
Prick to prick test with white and brown raw mushroom ( *Agaricus bisporus* )

Prick to prick test was positive to raw (9mm) and cooked (11mm) white *A. bisporus* , and raw (14mm) and cooked (11mm) brown *A. bisporus* ( Figure 1 ).

**Figure 2:**
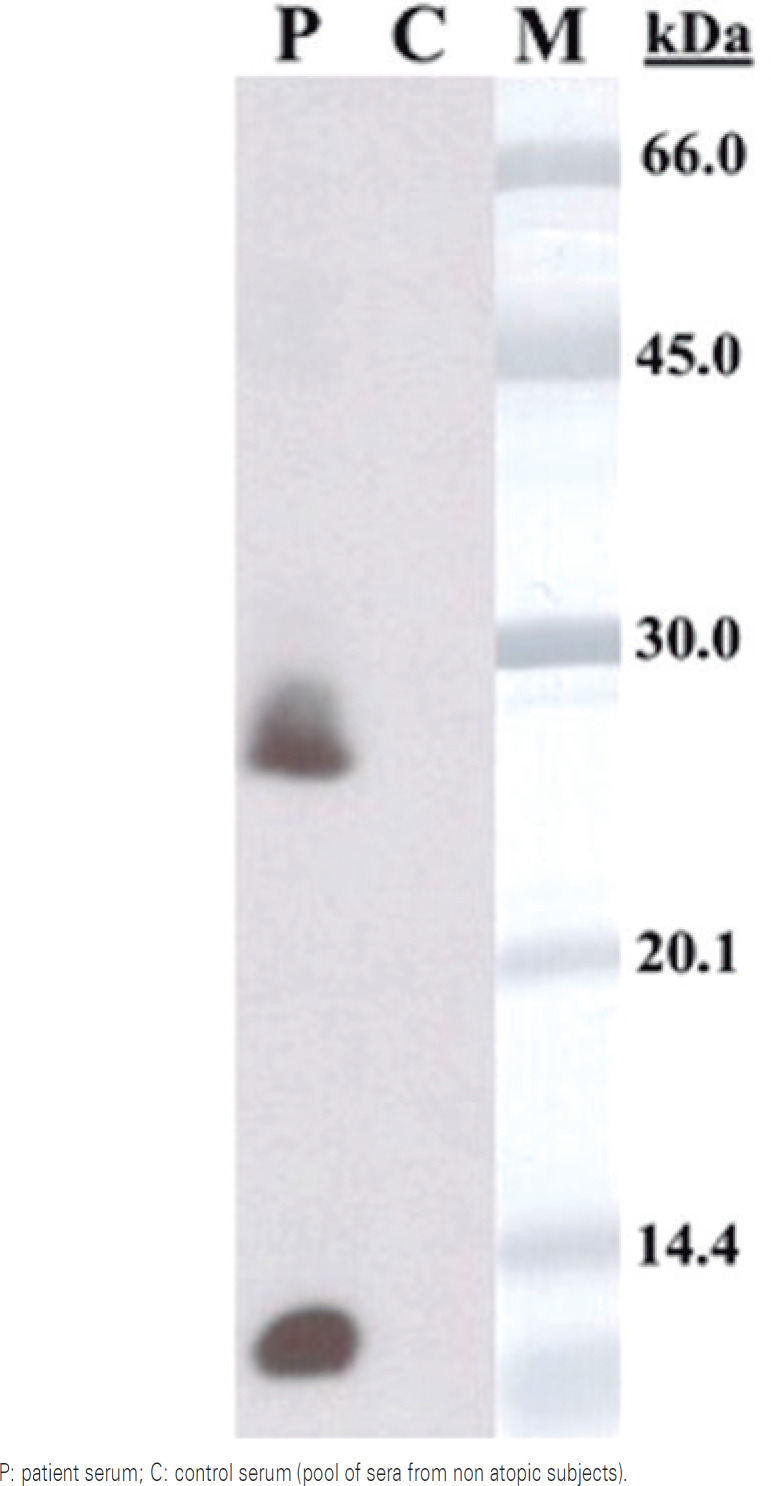
Sodium dodecyl sulfate-polyacrylamide gel electrophoresis immunoblotting results, band with mushroom extract

The immunoblotting assay with *A. bisporus* extract revealed two main IgE binding bands of approximately 10kDa and 27kDa ( Figure 2 ).

## DISCUSSION

In the literature there are a few cases of mushroom food allergy, most of them occurred in China, where mushrooms are included in the diet and the last known report was published in 2013.^(^^9^^-^^11^^)^

We report a case of anaphylaxis due to *A. bisporus* allergy in a patient with asthma and rhinitis, an uncommon case highlighting the importance of being aware of unsuspected food allergens.

According to skin tests and serum measurements, the patient was allergic to house dust mites and mushroom. The skin tests were negative to molds and all other suspected food allergen sources. Without mold sensitization, this patient probably presents a primary sensitization to mushroom by ingestion, and not a cross reactivity with molds or mushroom spores. The cases reported in the literature associated mushroom food allergy to cross reactivity: Carrapatoso et al. described a case of a young man with allergic rhinitis allergic to *A. alternata* , who had one episode of exercise-induced anaphylaxis after ingestion of *A. bisporus* ; Dauby et al. described a woman with allergic rhinitis, allergic to *Hormodendrum cladosporioides* , *A. alternata* , *Fusarium vasinfectum* , *Helminthosporium interseminatum* and to *Epicoccum nigrum* , with an oral allergy syndrome to raw *A. bisporus,* and in both case reports the authors believed the hypersensitivity reaction was attributable to cross reactivity between mold and mushroom allergens.^(^^11^^,^^12^^)^

Two thermostable IgE-reactive proteins with approximately 10kDa and 26kDa were detected, although protein class was not established, and these two allergens were unknown until our case report. In other case reports other mushroom allergens were identified. Hegde *et al* . were able to identify mannitol, the major carbohydrate component in *Fungi* , as one of the possible allergens of *A. bisporus* in a woman who had anaphylactic episodes, after ingestion of *A. bisporus* and pomegranate.^(^^13^^)^ Dauby *et al* . identified thermolabile *A. bisporus* proteins with molecular weight of 43kDa to 67kDa, which seemed to cross react with aeroallergens from mold, and were involved in a case similar to oral allergy syndrome.^(^^12^^)^

After the diagnosis of mushroom allergy, the patient was advised to eliminate mushroom and mushroom-containing products from his diet. He was prescribed an adrenaline autoinjector (Anapen 300ug/0,3mL) to use, if necessary.

## CONCLUSION

There are few described cases of mushroom food allergy, and the majority are due to cross-reactivity between molds and food allergens. The scarce data related to primary sensitization to edible mushrooms and food allergy became our diagnostic approach difficult, but make our results even more important, since our patient has a probable primary sensitization to *A. bisporus* . The first episode described by the patient fulfills clinical criteria for anaphylaxis, which is rather uncommon presentation. The immunoblotting assay revealed two IgE-reactive proteins with approximately 10kDa and 27kDa.

Although a food oral challenge was contraindicated, the test results and clinical history were essential to make diagnosis. The importance of considering less common allergenic sources as possible culprits is challenging, but it is of utmost relevance in the field of food allergy.
