# Supplementary material for: Anaphylaxis to Agaricus bisporus ingestion
Source: Einstein (Sao Paulo). 2020 Nov 18;18:eRC5478. doi: 10.31744/einstein_journal/2020RC5478 (PMC7687914; doi:10.31744/einstein_journal/2020RC5478)
Supplement: Translation

# Translation: Anafilaxia por ingestão de *Agaricus bisporus*

**Authors:** Inês Machado Cunha, Maria Luís Marques, Carmo Abreu, Borja Bartolomé, Eva Gomes

**Affiliations:** 1 Porto Portugal Serviço de Imunoalergologia, Centro Hospitalar Universitário do Porto, Porto, Portugal.; 2 Vila Real Portugal Serviço de Imunoalergologia, Centro Hospitalar de Trás-os-Montes e Alto Douro, Vila Real, Portugal.; 3 Bilbao Spain Research and Development Department, Roxall España, Bilbao, Spain.

**Keywords:** Anafilaxia, Agaricales, Immunoblotting

## Abstract

Paciente do sexo masculino, 33 anos, com asma e rinite alérgica desencadeadas por ácaros, relatou episódio de angioedema facial e labial, dispneia, tosse e disfagia aos 25 anos, minutos após a ingestão de uma pizza de cogumelo ( *Agaricus bisporus* ). O paciente negou consumo de medicamentos, picadas de himenópteros, ou quaisquer outros possíveis desencadeadores ou cofatores que pudessem estar presentes. Desde então, evita todos os tipos de cogumelos, até a ocorrência de um contato acidental com molho de cogumelo, que resultou em angioedema labial minutos após. O estudo imunoalergológico incluiu doseamento de IgE total e específica para cogumelos, testes cutâneos para aeroalérgenos, possíveis alérgenos alimentares e extrato de cogumelos, teste *prick to prick* com *A. bisporus* cru e cozido e teste de SDS-PAGE *immunoblotting* . O estudo revelou IgE específica para cogumelos de 0,76kUA/L, teste cutâneo positivo para extrato de cogumelos e teste *prick to prick* positivo para *A. bisporus* branco e castanho (cru e cozido). O *immunoblotting* identificou duas proteínas de ligação de IgE, de 10kDa e 27kDa. Relatamos, assim, um caso de anafilaxia por ingestão de *A. bisporus* , provavelmente explicado por sensibilização primária a cogumelos. Detectamos duas proteínas IgE-reativas de 10kDa e 27kDa como os possíveis alérgenos responsáveis.

## INTRODUÇÃO

A anafilaxia é uma reação alérgica grave, com início rápido e desfecho potencialmente fatal.^(^^1^^)^

Em adultos, os principais desencadeadores de reações anafiláticas são alimentos, picadas de insetos e fármacos.^(^^2^^)^

Em relação aos alérgenos alimentares, os principais desencadeadores identificados são amendoim, peixe, crustáceos, frutos secos e frutas frescas, principalmente em pacientes alérgicos a pólen; no entanto, temos que estar cientes de que os alérgenos implicados podem mudar conforme os diferentes padrões alimentares.^(^^3^^,^^4^^)^Em casos suspeitos de alergia alimentar, a história clínica, combinada a um diário alimentar, é extremamente importante para a identificação de alérgenos alimentares menos comuns.

Espécies do reino Fungi podem causar diferentes tipos de sintomas alérgicos. Alérgenos dos esporos de bolores são predominantemente associados a alergias respiratórias. Espécies de cogumelos podem estar implicadas em dermatite de contato e causar também sintomas digestivos, que correspondem a 1% das alergias a cogumelos.^(^^5^^,^^6^^)^

*Agaricus bisporus* é a espécie mais cultivada de cogumelos e representa 38% da produção mundial.^(^^7^^,^^8^^)^Foram descritos apenas raros casos de alergia por ingestão de *A. bisporus* . A maioria dos casos relatados de alergia após ingestão de cogumelos referem-se às espécies *Boletus edulis, Boletus badius, Lentinus edulus e Tricholoma matsutake.* ^(^^9^^,^^10^^)^

Relatamos aqui um caso de anafilaxia por ingestão de *A. bisporus* num paciente sem qualquer outra sensibilização a fungos, em que a ingestão parece ser a via de sensibilização primária.

## RELATO DE CASO

Indivíduo do sexo masculino, 33 anos, com asma e rinite alérgica, em tratamento com corticosteroides inalatórios e nasais e com alergia conhecida a *Dermatophagoides pteronyssinus* e *Dermatophagoides farinae* .

Apresentou reação anafilática aos 25 anos, com angioedema facial e labial, dispneia, tosse e disfagia alguns minutos após ingestão de pizza de cogumelo. Não havia qualquer outro desencadeador presente, como fármacos ou picadas de insetos. O paciente também negou ter realizado exercício físico ou consumido álcool nas horas que antecederam o episódio. Desde então, o doente evita cogumelos ou qualquer alimento contendo cogumelos.

O paciente relatou dois episódios posteriores de angioedema labial, sem nenhum outro sintoma, após contato acidental com molho de cogumelo.

Foram realizados doseamentos séricos de IgE total (ImmunoCAP™, Phadia, ThermoFisher Scientific, Uppsala, Suécia) e IgE específico (ImmunoCAP™, Phadia, ThermoFisher Scientific, Uppsala, Suécia) para aeroalérgenos ( *Dermatophagoides pteronyssinus* , *D. farinae* , *Lepidoglyphus destructor* , faneras de gatos e cães), bolores ( *Aspergillus fumigatus* e *Alternaria alternata* ) e cogumelos.

Realizamos também testes cutâneos com extratos comerciais (LETI Laboratories, Madrid, Espanha) de *D. pteronyssinus* (100HEP/mL); *D. farinae* (100HEP/mL); *L. destructor* (10HEP/mL); pólen de *Olea europaea* (30HEP/mL), *Corylus avellana* (30HEP/mL) e *Platanus occidentalis* (30HEP/mL); faneras de gatos e cães (30HEP/mL); *A. fumigatus* (150ug proteína/mL); *A. alternata* (30HEP/mL); *Cladosporium herbarum* (150ug de proteína/mL); *Candida albicans* (125ug de proteína/mL); pólen de gramíneas (30HEP/mL); leite de vaca (1.600ug de proteína/mL); ovos (1.400ug de proteína/mL); farinha de trigo (1.900ug de proteína/mL); tomate (10HEP/mL) e cogumelos (10HEP/mL). A histamina foi usada como controle positivo (10mg/mL).

Além dos testes cutâneos com extratos disponíveis comercialmente, foi realizado teste *prick to prick* com *A. bisporus* cru e cozido.

Foi efetuado *immunoblotting* por eletroforese em gel de poliacrilamida com dodecil sulfato de sódio (SDS-PAGE) para avaliar a massa molecular das proteínas de ligação ao IgE do *A. bisporus* .

## RESULTADOS

A IgE total foi de 240kU/L e a IgE específica foi positiva para cogumelos (0,76kU_A_/L), *Dermatophagoides peteronyssinus* (30,90kU_A_/L) e *L. destructor* (2,33kU_A_/L).

Os testes cutâneos foram positivos para extrato de cogumelos (7mm), *D. pteronyssinus* (10mm), *D. farinae* (8mm) e *L. destructor* (7 mm).

O teste *prick to prick* foi positivo para *A. bisporus* branco cru (9mm) e cozido (11mm) e *A. bisporus* castanho cru (14mm) e cozido (11mm) ( Figura 1 ).

**Figura 1:** Testes *prick to prick* para cogumelo ( *Agaricus bisporus* ) branco e marrom cru

O teste de *immunoblotting* com extrato de *A. bisporus* revelou duas bandas principais de ligação com IgE, de aproximadamente 10kDa e 27kDa. ( Figura 2 ).

**Figura 2:** *Immunoblotting* por eletroforese em gel de poliacrilamida com dodecil sulfato de sódio, faixa de resultados com extrato de cogumelos

## DISCUSSÃO

Na literatura, há poucos casos de alergia alimentar a cogumelos, os quais são relatados principalmente na China, onde os cogumelos fazem parte da dieta. O último relato conhecido foi publicado em 2013.^(^^9^^-^^11^^)^

Relatamos aqui um caso de anafilaxia por alergia a *A. bisporus* num paciente com asma e rinite. Trata-se de um caso incomum, que destaca a importância de estarmos alertas para alérgenos alimentares incomuns.

De acordo com testes cutâneos e dosagens séricas, o paciente era alérgico a ácaros e cogumelos. Os testes cutâneos foram negativos para fungos e para todos os outros alérgenos alimentares considerados suspeitos. Sem sensibilização a fungos, esse paciente provavelmente apresentava sensibilização primária a cogumelos por ingestão e não reatividade cruzada com esporos de bolores ou outros fungos. Os casos relatados na literatura associam alergia alimentar a cogumelos com reatividade cruzada: Carrapatoso et al. descreveram o caso de um jovem do sexo masculino com rinite alérgica desencadeada por *A. alternata* , que apresentou episódio de anafilaxia induzida por exercício após ingestão de *A. bisporus* ; Dauby et al. descreveram uma paciente do sexo feminino com rinite alérgica e alergia a *Hormodendrum cladosporioides* , *A. alternata* , *Fusarium vasinfectum* , *Helminthosporium interseminatum* e *Epicoccum nigrum* , com síndrome alérgica oral a *A. bisporus* cru – em ambos os relatos de caso, os autores associaram a reação de hiperssensibilidade à reatividade cruzada entre alérgenos de bolores e cogumelos.^(^^11^^,^^12^^)^

Duas proteínas IgE-reativas termoestáveis de aproximadamente 10kDa e 26kDa foram detectadas, embora a classe das proteínas não tenha sido estabelecida, e esses dois alérgenos fossem desconhecidos até a data do nosso relato de caso. Em outros relatos de casos, alérgenos de cogumelos diferentes foram identificados. Hegde et al. identificaram o manitol, principal componente carboidrato dos *Fungi* , como um dos possíveis alérgenos do *A. bisporus* numa paciente do sexo feminino, com episódios de anafilaxia após ingestão de *A. bisporus* e romã.^(^^13^^)^Dauby et al. identificaram proteínas termolábeis de *A. bisporus* com peso molecular de 43kDa a 67kDa, com suposta reatividade cruzada com aeroalérgenos de fungos e relacionadas a um caso semelhante a uma síndrome alérgica oral.^(^^12^^)^

Após o diagnóstico de alergia a cogumelos, o paciente foi instruído a eliminar da dieta cogumelos e produtos que os continham. Foi prescrito um autoinjetor de adrenalina (Anapen 300ug/0,3mL) para ser usado, se necessário.

## CONCLUSÃO

Há poucos casos descritos de alergia alimentar a cogumelos, e a maioria é explicada pela reatividade cruzada entre fungos e alérgenos alimentares. Os dados escassos relativos à sensibilização primária a cogumelos comestíveis e à alergia alimentar dificultam nossa abordagem diagnóstica, mas tornam nossos resultados ainda mais importantes, já que nosso paciente apresenta provável sensibilização primária a *A. bisporus* . O primeiro episódio descrito pelo paciente atende aos critérios clínicos de anafilaxia, uma apresentação bastante incomum. O teste de *immunoblotting* revelou duas proteínas IgE-reativas, de aproximadamente 10kDa e 27kDa.

Apesar da contraindicação do teste de provocação oral, os resultados dos outros testes e a história clínica foram essenciais para que se estabelecesse o diagnóstico. A importância de se considerarem fontes alergénicas menos comuns como possíveis responsáveis traz desafios, mas é essencialmente relevante no campo das alergias alimentares.
